# *In Situ* Surface-Enhanced Raman Spectroscopy of Cellular Components: Theory and Experimental Results

**DOI:** 10.3390/ma12091564

**Published:** 2019-05-13

**Authors:** Mario D’Acunto

**Affiliations:** IBF-CNR, Istituto di Biofisica, Consiglio Nazionale delle Ricerche, Area della Ricerca CNR di Pisa, via Moruzzi 1, I-56124 Pisa, Italy; mario.dacunto@pi.ibf.cnr.it; Tel.: +39-050-315-2763

**Keywords:** Surface-enhanced Raman spectroscopy, plasmonic Green function, gold nanoshells AuNSs, quasi normal modes

## Abstract

In the last decade, surface-enhanced Raman spectroscopy (SERS) met increasing interest in the detection of chemical and biological agents due to its rapid performance and ultra-sensitive features. Being SERS a combination of Raman spectroscopy and nanotechnology, it includes the advantages of Raman spectroscopy, providing rapid spectra collection, small sample sizes, characteristic spectral fingerprints for specific analytes. In addition, SERS overcomes low sensitivity or fluorescence interference that represents two major drawbacks of traditional Raman spectroscopy. Nanoscale roughened metal surfaces tremendously enhance the weak Raman signal due to electromagnetic field enhancement generated by localized surface plasmon resonances. In this paper, we detected label-free SERS signals for arbitrarily configurations of dimers, trimers, etc., composed of gold nanoshells (AuNSs) and applied to the mapping of osteosarcoma intracellular components. The experimental results combined to a theoretical model computation of SERS signal of specific AuNSs configurations, based on open cavity plasmonics, give the possibility to quantify SERS enhancement for overcoming spectral fluctuations. The results show that the Raman signal is locally enhanced inside the cell by AuNSs uptake and correspondent geometrical configuration generating dimers are able to enhance locally electromagnetic fields. The SERS signals inside such regions permit the unequivocal identification of cancer-specific biochemical components such as hydroxyapatite, phenylalanine, and protein denaturation due to disulfide bonds breaking between cysteine links or proline.

## 1. Introduction

Raman spectroscopy is a vibrational spectroscopic technique able to identify molecular species in a wide range of analytical applications [1]. One critical aspect of Raman spectroscopy is its relatively weak signals. Consequently, in the last decades, methods for enhancing the signal have been developed in order to enable the detection of small quantities of analytes [2,3,4]. The most efficient method to enhance the Raman signal involves the plasmonic confinement of electromagnetic fields due to metal nanorough surfaces [5]. This method takes the name surface-enhanced Raman spectroscopy (SERS) [6]. The strong Raman signal, generated by the SERS effect, enables short acquisition times consistent with the general requirements for both higher-throughput analysis and biological sampling and imaging [7]. When a molecule is located between two metallic nanoparticles (NPs), for example, both the fluorescence and the Raman cross section are much larger than for a molecule far from the metallic NPs. The enhancement factor is generally composed of two contributions: one is the electric field enhancement factor, Γ(*ω*) = |***E***|^2^/|***E***_0_|^2^, where ***E***_0_ is the incident electric field and ***E*** the electric field scattered at the position of the molecule. The other one, |Γ*_d_*|, is the enhancement factor that measures how much the decay rate of an excited state of the molecule is enhanced near the metallic nanoparticles. Since the SERS effect increases the Raman cross section by a factor ~|Γ|^2^, the fluorescence cross section increases by a factor of ~|Γ|^4^/|Γ*_d_*|^2^. For moderate to large molecule–particle distances, Γ(*ω*) and |Γ*_d_*| are mostly equal, on the contrary, for distances less than few nm |Γ*_d_*|, and can exceed Γ(*ω*). Thus, surface enhancement of fluorescence is much less marked than that of Raman scattering. 

In biology, SERS spectroscopy has been used to map fixed [8] or living cells [9] containing colloidal metal nanoparticles. Many studies have been addressed to exploit the full potential of this technique achieving the ability to manipulate cells in order to perform the Raman mapping on specific sites, or controlling the degree of enhancement to quantify SERS spectra features [10].

The investigation of cells by colloidal metal NPs-based SERS is made with two possible methods, label-based or label-free methods [11]. The label-based methods exploit functionalized nanoparticles (normally silver or gold) with specific biomarkers that can produce distinct and ultrasensitive signals. This method is limited in that while is able to provide the signatures of the biomarkers employed, it loses all the other rich information of the cells under investigation [12,13]. The accuracy of the label-based SERS method depends essentially by the bio-marker used. Contrary to the label-based SERS method, the label-free SERS method can detect cell components measuring the intrinsic SERS patterns of the cell in a faster and easier way. 

Since the first use of colloidal gold NPs for the observation of biological molecules by Kneipp et al. [14], the delivery of NPs into living or dried cells has achieved a sophisticated approach, depending on the nature of the experiments, to probe subcellular components [15]. However, some critical questions should be still addressed. Metal NPs enter cells by endocytosis. This process involves several pathways [16]. Once inside cells, metal NPs are often clustered and distributed non-uniformly producing significant variations in SERS spectra because the SERS signal takes place prevalently in the local fields generated between the metal NPs. Together to SERS signal, the metal NPs play a role in the rise of background and fluorescent signal [17]. The fluorescence background takes place in correspondence with enhanced fields by the metal NPs hiding spectral information and making it difficult to achieve unequivocal conclusions in the case of inhomogeneity distributions of metal NPs. Another critical question is represented by the difficulty in comparing spectra of different spots inside the cells. This is because, generally, the enhancing fields are quantitatively unknown and fluctuations in the scattering signals can be observed at relatively higher laser powers [18], when SERS enhancement factors can be of the order ~10^4^–10^6^. In addition, fluctuations and reduction of spectral intensity of scattering signals in SERS measurements can be generated by thermal fluctuations induced by heating effects of the highly confined enhanced fields between metal NPs. All such critical questions suggest that, in principle, there is no way to infer the number of molecules contributing to SERS signal from its absolute intensity.

In this paper, we introduce numerical SERS enhancement maps depending on the arrangement of the AuNSs internalized in the upper layers of the hosting cells. We demonstrate that in complex environments like inner mammalian cells, when the spatial maps of the SERS enhancement are known, it is possible to have a reasonable estimation on the average number of molecules in the scattering volume of the SERS active areas defined by the AuNSs clusters. Although preliminary and qualitative, the advantages of our results in the application of SERS spectroscopy in biology and oncology are straightforwardly understandable.

## 2. Green’s Function Description of SERS Effect Generated by AuNSs

The main features of SERS spectroscopy lie in its ability to enhance the electromagnetic signal stimulated by the hotspots in nanostructured metals. The enhancement factor can be defined as the ratio of SERS signal to the Raman signal that would be obtained for the same substance in the absence of the SERS substrate, with all the other conditions being similar. Since differential Raman cross-sections can vary significantly, spanning a range *d*σ/*d*Ω~10^–30^–10^−24^ *cm*^2^, depending on molecule type, excitation wavelength, and resonance conditions [19]. An operative definition of SERS enhancement is, hence, *EF* = (*I*_SERS_/*N*_surf_)/(*I*_RS_/*N*_vol_), where *I*_SERS_ is the SERS intensity, *I*_RS_ the intensity for non-SERS Raman measurement. A more accurate definition of the Raman scattering molecules requires that *N*_surf_ be the number of molecules located in the hotspot region, typically confined to a surface rather than a volume, and *N*_vol_ is the average number of molecules in the scattering volume involved in the non-SERS Raman measurements [19,20]. This definition of enhancement factor requires that the number of molecules inside the hotspots is accurately determined. Since an accurate determination of the number of subcellular components is currently not possible, our approach is to determine precisely the enhancement factor and its spatial distribution. The critical question on the complete knowledge of the electric field, and hence of the enhancement factor, can be addressed in the quantification of the Green function of the metal–insulator–metal (MIM) system composed by two or more AuNSs close one each other. To do this, consider *N* identical dipolar spherical AuNSs of total radius *R*, polarizability *α*, located at the positions ***r****_i_* and an incident monochromatic light characterized by a complex electric-field amplitude ***E***_0_(***r***, *ω*). The size of the AuNSs is supposed to be small compared to the wavelength of the incoming light and, for sake of simplicity, the electric permeability and the polarizability of the AuNSs are assumed isotropic. For more details, see Appendix A. 

The detected intensity is proportional to the spectral density that is averaged square modulus of the electric field, i.e., $I\left(r_{0},\omega \right)\propto \langle E^{*}\left(r_{0},\omega \right)E\left(r_{0},\omega \right)\rangle$, being the detector located at ***r***_0_, so that the problem consists in calculating the electric field amplitude at each particle position *i*. Since all the AuNSs are in mutual interaction one each other, the problem is self-consistent and Green’s dyadic tensor formalism is the best choice to solve the problem. The mutual external electric field ${Ε}_{i}^{ex}\left(\omega \right)$ is expressed by the relation

(1) $$E_{i}^{ext}\left(\omega \right)=E_{0}\left(r_{i}\right)+\sum_{j\neq i}\alpha G\left(r_{i},r_{j},\omega \right)\cdot E_{0}\left(r_{j}\right)$$

where ***G*** is the Green dyadic function of the complete system. Once the external electric field is known at each position ***r****_i_*, the total electric field can be calculated

(2) $$E\left(\omega \right)=E_{0}\left(r\right)+\sum_{j=1}^{N}\alpha G\left(r,r_{j},\omega \right)\cdot E_{j}^{ext}$$

so that the detected intensity can be calculated. The second components in Equation (2) is given by the dyadic Green’s function that accounts, among other factors, the atomic roughness in the MIM cavity [21]. Since the system can be considered as a rough surface, which roughness asperities are composed by the AuNSs, we model the profile *z* = *h*(***x***), where we are using the notation that ***x*** = ***x′*** *x* + ***y′*** *y*, with ***x′*** and ***y′*** being unit vectors along the *x* and *y* directions. We can generalize and employ the analytical expression developed for rough surfaces [21], which Green function reads

(3) $$G\left(r,r_{j},\omega \right)=\frac{\delta }{k_{z}{\left(\partial \Theta /\partial k\right)}_{k_{r}}}H_{0}^{1}\left(\sqrt{\frac{\varepsilon_{m}\left(\omega \right)}{1+\varepsilon_{m}\left(\omega \right)}}kR\left(k\right)\right)$$

where $H_{0}^{1}$ denotes the Hankel function of first kind and order zero, *ε_m_*(*ω*) represents the metal electric permittivity described by the Drude model Equation (13) (Appendix A), *k_r_* is a spatial wavevector being the root of Θ(*k_r_*)+1 = 0 (where Θ is a function linking the reflection coefficient to spectral density and plasmon wavevector, see [21]), in turn, *δ* is a numerical factor. The singularities in Equation (3) correspond to the eigenmodes driving the plasmon resonance and the field enhancements. This is because the pole accounts for all the most relevant contributions to Green’s functions from the random rough surface. 

The non-linear nature of Equation (3) implies the advantage to have spatially narrow electromagnetic enhancements, narrower, in particular, of laser-beam spot size. This property results in a *super-resolution* SERS effect able to discriminate, as we will show, single intracellular components. This characteristic of AuNSs takes advantage on the spatial properties of hotspots generated by AuNSs clusters, dimers, trimers, etc. The AuNSs clusters correspond to metal–insulator–metal (MIM) nanogaps, which are open nanocavities able to trap the light at the cavity mode frequencies. The light-matter interaction properties of such nanogaps, their hot spots and their dependence of light polarization can be conveniently described in terms of the resonant cavity quasinormal modes (QNMs) [22]. In Appendix B, the reader can find a more accurate description on the physical nature of QNMs, here we are interested to enlist their deep connection with local density of states (LDOS) inside the open cavity generated by the AUNSs. LDOSs represent the weight of all normalized QNMs at a certain point of space for a certain light frequency and can be calculated by the relation:

(4) ρ(ωn,r)=2π2ωnIm[n⋅G(ωn,r,r′)⋅n′]≅1Δωn|n′⋅E(r′)|2∫dVε″|E|2

where ***G*** is the Green tensor, Δ*ω*_n_ is the spectral width of the mode and |***n⋅E***(**r**)|^2^ = *I*(**r**) its local intensity. So ultimately, in the picture of MIM cavity, only the cavity *Q*-factor and the effective mode volume *V_eff_* can essentially characterize the nanogaps generated by the AuNSs, so that a large *Q/V_eff_* ratio results in enhanced light-matter interactions as typically quantified by the LDOSs. We can say that the combination of applied polarization of the electric fields and the geometrical AuNSs arrangement introduces a sort of SERS *super-selection rules* on the Raman signal and the enhanced biochemical components (see Appendix C). 

Analogously to a Raman imaging map, also SERS map can be regarded as a matrix ***X*** of dimension *m* by *n*, where the Raman spectrum of each recorded position correspond to a row vector, the spectra modified by SERS effect can be linked to spatial hotspots. Hence, since a Raman map described by ***X*** matrix is

(5) $$X=\left|\begin{array}{cccc} x_{1}\left(1\right) & x_{1}\left(2\right) & \ldots & x_{1}\left(n\right)\\ x_{2}\left(1\right) & x_{2}\left(2\right) & \ldots & x_{2}\left(n\right)\\ \vdots & \vdots & \vdots & \vdots \\ x_{m}\left(1\right) & x_{m}\left(2\right) & \ldots & x_{m}\left(n\right) \end{array}\right|$$

where *m* is the number of spectra traces performed and *n* is the number of data points per spectrum along the wavenumber axis, respectively, the SERS maps enable a sub-data set inside ***X*** with a restricted number of spectra that can be put in correspondence with the spatial localization of hotspots generated by the AuNSs. The matrix ***X***(*m* × *n*) can be considered as a matrix product

(6) X=WH

where ***W*** is a *m*×*p* matrix whose columns represent pure component spectra and ***H*** is a *p*×*n* matrix whose rows represent intensity profiles of corresponding pure spectral components. The parameter *p* generally defines the number of components determined a priori during the data processing. However, although an appropriate number of components can be estimated by using multivariate analysis, principal component analysis or singular value decomposition, for example, the opportune *p* number in decomposing spectral components for biological sample can be a complicate challenge. The SERS map can help to define a suitable *p* number components. This is because the factor |Γ|^2^ reduces the complexity of data to small low-rank matrix ***X***′ ≠ 0 determined essentially by the SERS effect, with all data points = 0. The correspondent new ***W***′ and ***H***′ are iteratively refined using alternating least squares so that matrix norm ||***X***′-***W***′***H***′|| is minimized. Analogously to ***W*** and ***H***, non-negative constraints on both ***W***′ and ***H***′ matrices are also imposed, since neither the Raman spectra nor its concentration profiles can take negative values in practical terms.

## 3. Materials and Methods

The present investigation is focused on how SERS signals relatively to mesenchymal stem cells (MSCs)-differentiated MG-63 osteosarcoma cells can identify chemical components in several subcellular areas.

First, MSCs were isolated from human bone marrow and plated on glass slides inside Petri dishes supplemented with 10% fetal bovine serum. The samples were cultured for 72 h, then fixed in 1% neutral buffered formalin for 10min at 4 °C.

Then, MG-63 (human osteosarcoma cell line ATCC® CRL-1427) cells were seeded on six slides at 10000 cells in Eagle’s minimum essential medium completed with 10% fetal bovine serum. The samples were cultured for 72 h, and then fixed in 1% neutral buffered formalin for 10 min at 4 °C. Thereafter, the cell samples, composed by glass slides containing a population of nearly 20000 cells, were loaded with AuNSs diluted 1:20 in the culture media 24 h before the endpoint. Hence, the samples were fixed in 1% (w/v) neutral buffered formalin for 10 min at 4 °C.

**Figure 1:**
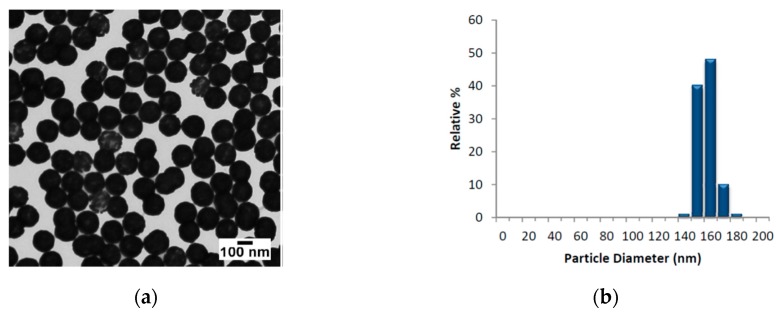
Transmission electron microscopy image of the gold nanoshells (AuNSs) (**a**); correspondent size distribution (**b**).

The AuNSs employed, provided by Nanocomposix@, were composed by nominal diameter of 152.7 ± 6.8 nm (core diameter 119.7 ± 8.8 nm). In Figure 1, we report the TEM images of AuNSs, the size distribution and the absorbance profile in the range 300–1100 nm, respectively.

Raman spectra were recorded with a Thermo Fisher Scientific DXR2xi Raman microscope. The experimental parameters were identified after preliminary tests performed to optimize the signal-to-noise ratio, and minimized the sample fluorescence. The best experimental set up were identified with the following parameters: Laser wavelength 532 nm; power laser of 8–10 mW; 400–2000 cm^−1^ Raman shift, 25 μm confocal pinhole; 5 (FWHM) cm^−1^ spectral resolution and a 100 × objective resulting in a laser spot size of approximately 700 nm. Integration time for recording a Raman spectrum was 1 sec and 10 scans for any spectrum. Raman spectra, to which was subtracted background, were normalized for the area under the curve in order to standardize the Raman intensities. Raman spectrometer is coupled to confocal microscope operating both in bright- or dark-field. Raman imaging was performed with steps of 0.1 μm for mapping and each pixel corresponds to one scan. With this experimental scanning setup, any Raman map takes several hours to be completed.

Multivariate techniques employed an unsupervised method, such as Principal Component Analysis (PCA) and a supervised method as Linear Discriminate Analysis (LDA) [23]. PCA is widely employed to simplify a complex dataset of multiple dimensions into a smaller set of orthogonal linear combinations to create uncorrelated variables known as principal components (PCs). The PCs maximize the variance in the data. The lowest-order PCs carry the greatest information and leading to restrict the number of PCs used in the subsequent classification algorithms. PCA was applied to X matrix (Equation (6), hence LDA was applied to find and classify possible differences among the populations of cells under investigation. Unlike PCA, which is an unsupervised method, LDA tries to maximize the data variation between different classes known a priori, for example, how each peak corresponds to specific cell populations. 

TEM images of AuNPs were obtained by using a JEOL-JEM-1400FLASH) JEOL ltd, Peabody, Massachusetts, USA) microscope. SEM images of cells were obtained by using a Phenom XL desktop microscope (Phenom-World, Eindhoven, The Netherlands).

## 4. Experimental Results

The experimental study was focused to exploit local enhanced fields generated by AuNSs in osteosarcoma cells for identifying biochemical components characterizing the cancer cells. Before application of SERS, Raman spectroscopy and imaging were carried out on osteo-differentiated MSCs and osteosarcoma cells with the aim of discriminating between the different the cell populations. The ability to discriminate between osteo-differentiated MScs and osteosarcoma cells consists in identifying the major biochemical components characterizing osteosarcoma cells. Hence, in the second experimental stage, SERS was applied to osteosarcoma cells with the aim of understanding if and how local fields generated by the AuNSs (previously internalized in the MG-63 cells) enhance the biochemical components characterizing the osteosarcoma cells. 

**Figure 2:**
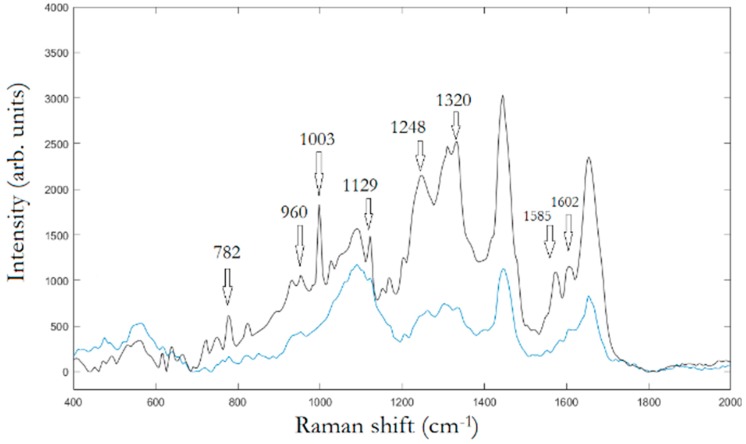
Averaged Raman spectra collected upon osteo-differentiated mesenchymal stem cells (MSCs) nucleus, blue line, and correspondent spectra collected upon MG-63 nucleus, black line.

In the first experimental step, Raman mapping was performed on 20 cells for any cell population. In Figure 2, the averaged Raman spectra of osteo-differentiated MScs and MG-63 cells, respectively, are reported.

**Figure 3:**
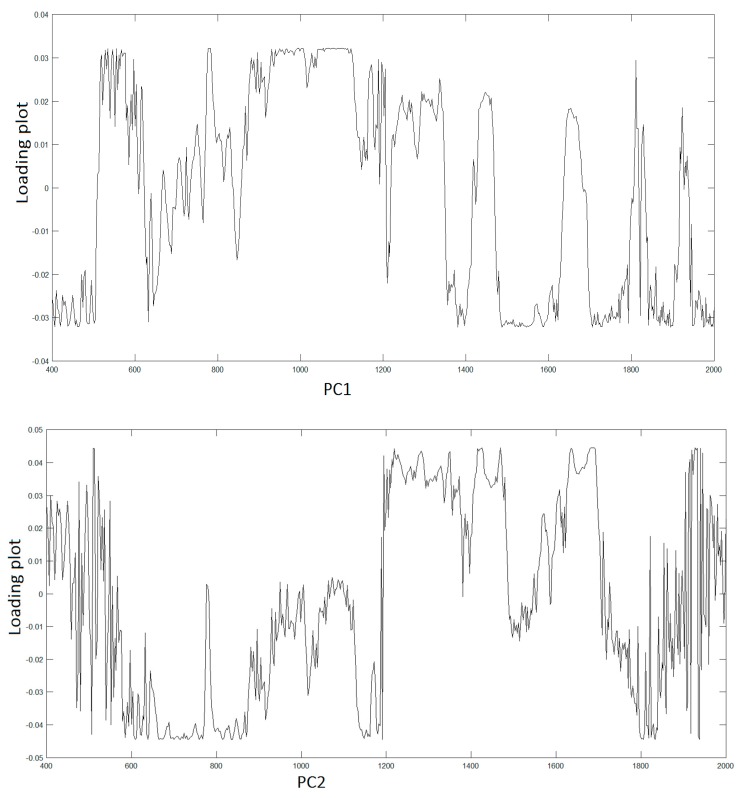
First two principal component (PC)1,2 loading plots.

To assign the peaks, we adopt PCA to extract the relevant information from the original data producing a new set of variables consisting in the PCs covering the 95% of the total variance of the raw data. Hence, LDA was used to find similarities and differences between osteo-differentiated MScs and MG-63 cells. All the information about the possible differences is contained within the first four PCs, PC1 (51,7%), PC2(23,8%), PC3(15,2%), and PC4(7,0%), respectively. Figure 3 displays the spectra of first two PC vectors summarizing main peaks of each PC that can be related to Raman bands of collected raw data.

Table 1 reports the main biochemical components present in MG-63 cells with no correspondence in osteo-differentiated MSCs.

**Figure 4:**
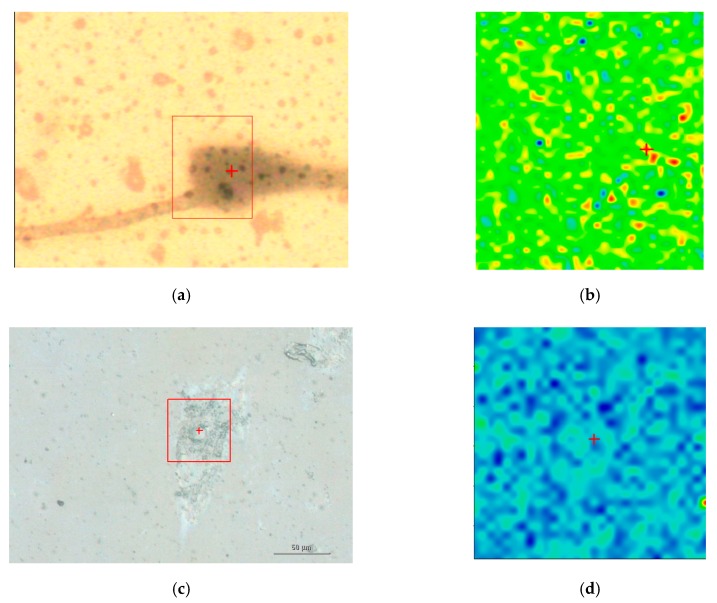
Raman chemical maps of a MG-63 cell and an osteo-differentiated MSC. The images a and c represent the cell morphologies with the red box denoting the area under Raman investigation for MG-63 (**a**) and osteo-differentiated MSC (**c**), respectively. The images A (area 30 × 40 μm^2^) and C (scale bar 50 μm) were recorded making use of 50× magnification. The images B and D represent the chemical Raman imaging for the MG-63 (**b**) and the osteo-differentiated MSC (**d**), respectively, reporting the correspondent distribution of HA (960 cm^−1^). Under the experimental setup adopted, the chemical Raman maps were collected with steps of nearly 1 μm.

Among the biochemical components that characterize the MG-63 cells compared to the osteo- differentiated MSCs, the hydroxyapatite (HA) plays a particular role, whose increase in MG-63 is an evidence to the degree of malignancy of MG-63 [23]. Vibrational spectroscopy can highlight the phosphate group (*PO*_4_)^3−^ that is a basic unit in HA, characterized by symmetric stretching (*P-O*) mode at 960 cm^−1^. Raman hyperspectral maps represented in Figure 4 display a greater amount of HA in MG-63 if compared to osteo-differentiated MSC cells, respectively. Indeed, the false colors yellow-red denote high concentrations of symmetric stretching (*P–O*) mode at 960 cm^−1^, on the contrary, blue color denotes low or very low presence of such mode of the phosphate group, and therefore lower levels of HA.

After multivariate analysis was applied and the major biochemical differences between osteo-differentiated MSCs and osteosarcoma cells were found, we shift the focus to local SERS signals. We were interested in the possible numerical quantification of subcellular components as revealed by the SERS signals, which, once the SERS signal was simulated by Equation (2) and matrix, Equation (6), was rescaled accordingly. In summary, experimental results have shown, that when compared to osteo-differentiated MSCs, osteosarcoma MG-63 cells show that components such as Cysteine, HA (a mineral form of calcium-phosphate) or Phenylalanine (Phe), are present in greater quantities. Rather than applying the method to study the biochemical differences between cell populations, in this paper, we demonstrate the identification of such characteristic components in situ in single cells focusing on osteosarcoma cells and showing how biochemical information can be obtained by local SERS mapping. 

Our approach can be summarized as follows. The first step of our approach is to have a map of the spatial extension and intensity of the hotspots generated by the AuNSs. The experimentally-measured Raman maps identify the hotspots generating the SERS signals. Essentially, the SERS map is a map of |Γ(*ω*)|^2^, where *ω* denotes the QNMs spectrum (see Appendix B) and the LDOS, Equation (4), and the correspondent Green function is experimentally available. *G_exp_* The QNMs are assigned to biochemical components located inside the enhancing hotspots. In addition, the identified hotspots define and localize the open cavities generated by the AuNSs. Hence, such hotspots enable the possibility to calculate the Green function (Equation (3)), enhanced electromagnetic fields (Equation (2)), and the correspondent QNMs spectra via the LDOS (Equation (4)) with the constraints of the experimental values previously collected. Once the new SERS map is calculated and the spatial distribution of enhancement factors are known, the Raman spectra of the SERS active domains are processed and compared in order to match the experimental spectra. In this way, the complete spectral mixtures correspondent to SERS domains are resolved and the possible subcellular components located inside the hotspots identified. 

**Figure 5:**
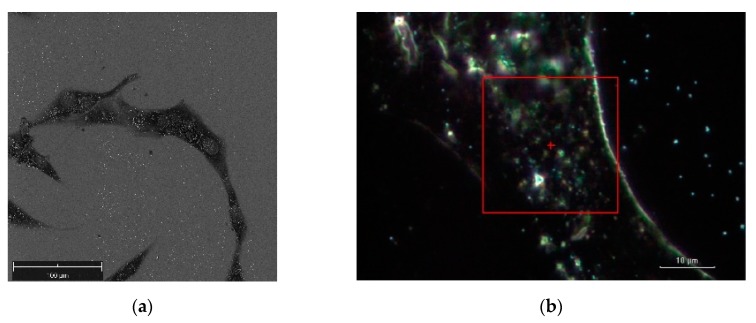
(**a**) SEM image of a cluster of MG-63 cells, the bright dots denote the AuNSs, scale bar 100 μm. (**b**) Dark-field illumination at 100× magnification on the DXR Raman microscope of a MG-63 cell crowded by gold nanoshells (AuNSs). The area under investigation is denoted by the red borders, scale bar 10 μm.

Figure 5 illustrates a SEM image and a dark-field illumination image of a MG-63 cell with AuNSs, respectively. 

**Figure 6:**
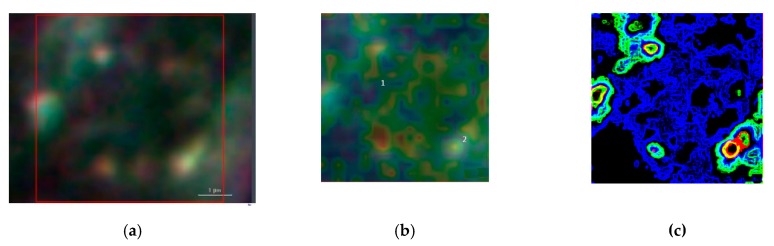

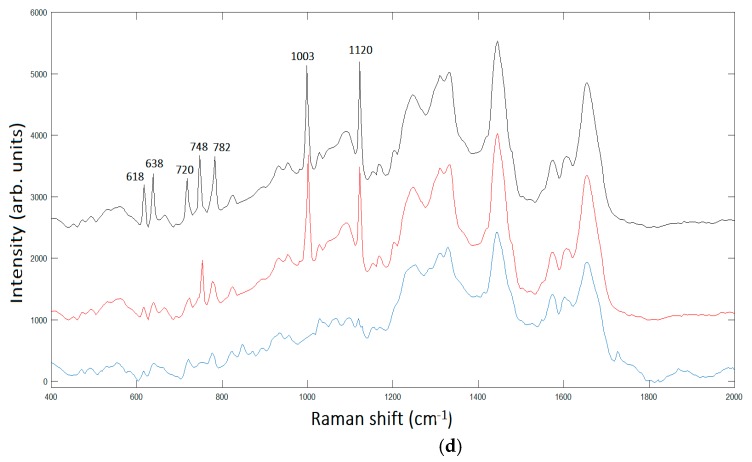
(**a**) Denotes the bright-field image of MG-63 nuclear area where the bright points display the AuNSs, scale bar 1 μm, (**b**) represents the correspondent Raman map of the area marked by the red box in (**a**). The labels 1 and 2 mark a non-SERS and a SERS domain characterized by the AuNSs dimer. (**c**) Shows the Raman map calculated with Green’s function: the red regions denotes the spatial areas with Γ^2^~10^5^. (**d**) Displays the Raman spectra in the recorded Raman map, label 1 (blue line), label 2 (red line) and in 2 after data processed with simulated map (**c**), black line.

Figure 6 summarizes the basic features of our method. It is displayed a zoom area with a dimer generating a SERS signal, denoted by 2 in Figure 6b and the correspondent Raman spectra with labelled peaks in Figure 6d. Figure 6a denotes the dark-field image of subcellular area under investigation. The bright points display the AuNSs. Figure 6b represents the correspondent Raman map. The labels 1 and 2 mark a non-SERS (blue line in Figure 6d, and a SERS domain (green line in Figure 6d), the latter characterized by the AuNSs dimer, respectively. Once the Raman map enables the identification the SERS active regions, an equivalent SERS map, a map of |Γ(*ω*, *θ*)|^2^, is calculated by using Equations (2) and (3) with the AuNSs configuration as in the correspondent optical image. The SERS map essentially shows the spatial distribution of enhancement factors |Γ|^2^~10^5^ as in Figure 6c. The knowledge of the spatial distribution of the enhancement factor implies the identification of the LDOS of the specific dimer under investigation, as given by Equation (4). LDOS contains the QNMs that represent a sort of pure component from which the Green function can be accurately calculated. After the SERS map is available, the spectral mixture is unmixed by modeling the spectra according to an iterative least squares-based algorithm with appropriate constraints and a priori assumption of pure components knowledge in Equation (6). The unmixing method can be described as follows. The SERS map is a hyperspectral image that is first unfolded to form a matrix denoted by ***X***′. ***X***′ is unmixed using *p*′ number of pure components (a number which has been previously identified by Raman map, (Figure 6b)) so that now ***H′*** is a matrix *p*′ × n, every row of which contains the spectra of a precomponent. By Equation (6), ***W***′ is obtained and it can be used to generate segmentation maps and corresponding centroid profiles. Analogously to Multivariate Curve Resolution algorithms, the segmentation maps require a number of clusters to describe chemically relevant zones in the sample [24]. Each cluster includes different contributions from any pure component weighed by the centroid profiles. 

The peak assignment in Figure 6d provides bands to 618 cm^−1^ corresponding to *C–C* twisting in proteins, 638 cm^−1^ corresponding to *C–C* stretch of Proline (Pro) ring [26]. In addition, we have three bands 720 cm^−1^, 748cm^−1^, and 782 cm^−1^, respectively, corresponding to DNA [24]. The enhancement of such peaks’ correspondent DNA is very reasonable, being the area under investigation located in the cellular nucleus. In turn, the Phe peak 1003 cm^−1^; and the peak at 1120 cm^−1^ corresponding to β-carotene are also present in the unprocessed spectrum [25]. It is interesting to note the contemporary signal enhancement of β-carotene and Pro. β-carotene is usually found in cancer cells, due to its role in apoptosis of cells. Apoptosis is a protective mechanism against neoplastic transformation and proliferation by eliminating genetically damaged cells. It has been demonstrated that β-carotene exhibits a potential role in the induction of apoptosis of human cervical cancer cells [27], colon adenocarcinoma [27], leukemic cells [27], gastric cancer cells [27], and papillary thyroid carcinoma [28]. The role of β-carotene in inducing apoptosis in osteosarcoma is still to be clarified. Endogenous Pro is synthesized mainly from Glutamine and can play an important role in cell signaling. Under normal metabolic regulation, Pro is incorporated into collagen, the most abundant protein in the body. Since a reservoir of Pro is a source of amino-acids and energy during conditions of metabolic stress, and is regulatory of apoptosis and autophagy, the presence of a certain amount of proline may be a measure of tumor malignancy [26]. 

One limitation of our approach is that the SERS signals were generated *in situ*, i.e., by the random localization of dimers or trimers of AuNSs inside the cells, although SERS signals presented a good level of reproducibility and accuracy. In addition, the simulation of SERS map arises a critical question arises about the unicity of the map for the specific 3D AuNSs configuration created by the AuNSs locations.

To overcome such questions, we have to take into consideration the *z*-axis location of the AuNSs. Inside the SERS active region, the Raman detector collects the scattered light of a cylinder of height ~700–800 nm, being the penetration depth of a beam at 532 nm approximately ~700–800 nm, and a base defined by the enhancement region between the AuNSs.

The relative permittivity of the surrounding medium must be taken into due consideration, being the inner cell environment denoted by the water dielectric constant joint to air out the cell. 

**Figure 7:**
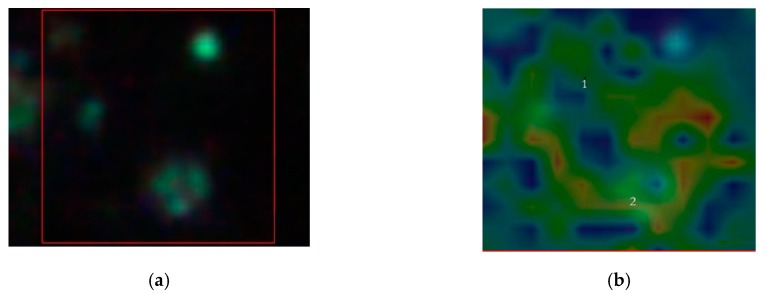

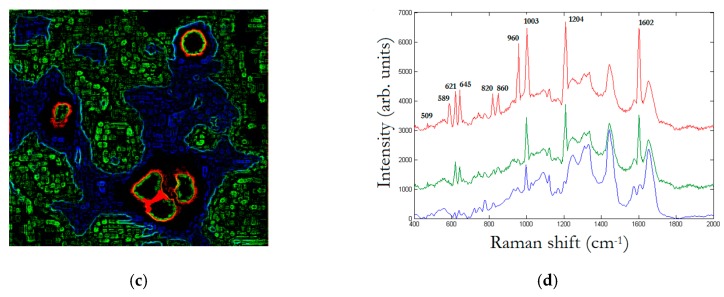
The labels (**a**), (**b**), and (**c**) as in Figure 6, in a nuclear area (5 μm × 5 μm) of a MG-63 cell, in Figure 6c red region denotes Γ^2^~10^5^. (**d**) Represents the correspondent spectra in 1 (blue line), 2 (green line), and in 2 after SERS map (red line).

In Figure 7, we replay the calculation for a trimer, whose SERS map reveals that the enhancement is limited to a dimer. The peak assignment in Figure 7d, as in Figure 6d, is performed through multivariate analysis, and provides a peak to 509 cm^−1^ which corresponds to *S*-*S* disulfide stretching in collagens, 589 cm^−1^ corresponding to symmetric stretching vibration of phosphate ($\nu_{4}PO_{4}^{3-}$) of HA, and 621 cm^−1^ and 645 cm^−1^ corresponding both to *C–C* twisting mode of Phe [23,25]. Other relevant peaks enhanced by the SERS effect regard structural protein modes of tumors at 820 cm^−1^, phosphate group at 860 cm^−1^, symmetric stretching vibration of $\nu_{1}PO_{4}^{3-}$ in HA at 960 cm^−1^, and Phe peak at 1003 cm^−1^. In turn, we have additional enhanced peaks at 1204 cm^−1^ corresponding to Amide III and CH_2_ wagging vibrations from Glycine and Proline side chains and at 1602 cm^−1^ corresponding to Phe, respectively [23,25]. In particular, we wish to remark the contemporary incidence of signals correspondent to S-S disulfide stretching in collagens and Amide III peak correspondent to Proline side chains demonstrating a possible metabolic mechanism of collagen denaturation [26]. 

The results shown in Figure 6 and Figure 7 demonstrate the possibility to remark local biochemical components in cancer cells. Such results obtained by exploiting the enhancement ability of SERS spectroscopy can have significant implications in the grading of tumors. Increases of HA, Phe, on one side, and the incidence of Proline signals correspondent to collagen tissues and the attenuation of S-S disulfide stretching signal, on the other side, can be considered both witnesses of the degree of malignancy of MG-63 cells or an evidence of protein denaturation processes [18,23,29].

In turn, a special advantage of our method based on the Green’s function is the quantification of heating effects in SERS maps. It is a common experience to destroy cells by heating when the AuNSs absorb a certain amount of energy transferred as heat. Since, due to light absorption, the AuNSs are the sources of heat, it should be noted that our theory gives the possibility of locally calculating the thermal energy generated around the AuNSs. From the absorbed power *Q*(***r****_i_*) = *σ_abs_I*, indeed, once the electric field distribution, Equation (2), is known, the heat power, *q*(***r****_i_*) = *Q*(***r****_i_*)/*V* (where *V* is the AuNSs nanostructured MIM volume) can be calculated as

(7) q(ri)=ωIm(αi(ω))|Ei|2

end, in the steady-state regime, the temperature intensity, *T*(***r***), throughout the cells follows the Poisson equation

(8) $$\kappa \nabla^{2}T\left(r\right)=-q\left(r\right)$$

By using Equation (2), we can write the temperature intensity in terms of the Green’s function, Equation (3), as [30]

(9) T(ri)=∑j=1NG(ri,rj)ωvIm(αj(ω))[κ2−κ1κ2+κ11|ri−rj|]T0

where *κ*_1_ denotes the thermal conductivity of cell environment, *κ*_2_ the thermal conductivity outside the cell (air) and *T*_0_ is the room temperature. For the experimental conditions adopted and considering a thermal conductivity of water for the inside of the cell environment as *κ*_1_ = 0.6 W/mK, and *κ*_2_ = 0.25 W/mK for air, the enhancement of the temperature is approximately 5–8 °C. 

## 5. Conclusions 

Raman spectroscopy, amplified by SERS NPs can provide imaging modality including high molecular specificity, high sensitivity, and negligible autofluorescence. The absorption of nanoparticles by the cells via endocytosis allows exploitation of the hotspots generated by the plasmonic properties of the NPs to perform internal spectroscopy of the cells themselves and to discriminate between healthy- and tumor cells. In this paper, firstly, experimental measurements were carried out on osteo-differentiated MSCs and osteosarcoma cells with standard Raman spectroscopy, respectively. Hence, we have focused on the numerical quantification of subcellular components of osteosarcoma cells, by using the SERS signals exploiting the AuNSs internalized in the MG-63 cells. The local SERS signal is simulated by Equation (2) and matrix Equation (6) is rescaled accordingly. We have shown that biochemical components characterizing osteosarcoma cells, such as hydroxyapatite, a mineral form of calcium-phosphate, Cysteine, or Phe, two essential α-amino acids, can be obtained by local SERS mapping. In addition, local SERS signals reveal new enhanced components providing a local spectroscopy analysis that can elucidate the biochemical transformations of cancer cells, such as Proline amino acid in its connection to the metabolism of collagen, or β-carotene in connection to the apoptosis mechanism of elimination of neoplastic cells. 

The methodology presented in this paper is general, requiring only an accurate experimental knowledge of the distribution of metallic nanoparticles and can be applied to all SERS methodologies in oncology.

## Figures and Tables

**Table 1:** Peak position and assignment of major Raman bands observed in MG-63 cells with respect osteo-differentiated MSCs. In bold are the bands characterizing the MG-63 cells. References [23,24,25].

Raman Shift (cm^−1^)	Band Attribution
668	*v*(*C-S*) **Cysteine**
782	**Ring breathing modes in DNA**, RNA bases
959–962	$v_{1}PO_{4}^{3-}$
1003	*v*(*C-C*) Phenylalanine
1035	$v_{3}PO_{4}^{3-}$ overlaps with proline components
1048	$v_{3}PO_{4}^{3-}$
1129	*v*(*C-C*) skeletal of acyl backbone in lipids
1176	v(C−O−C), Tyrosine, **Phenylalanine**
1204	$\omega \left(CH_{2}\right)$ Tyrosine, hydroxyproline
1248	*NH*_2_ Guanine, Cytosine DNA, RNA
1272	Amide III, protein *α*-helix
1293–1305	δ(=CH)
1320	*CH* deformations, proteins
1585	v(C−C−H)
1602	Phenylalanine

## Abbreviations

SERS	Surface-Enhanced Raman Spectroscopy
AuNS(s)	Gold Nanoshell(s)
NP	NanoParticle
MIM	Metal-Insulator-Metal
QNM	QuasiNormal Mode
LDOS	Local Density of States
MSC	Mesenchymal Stem Cells
HA	HydroxyApatite
Phe	Phenylalanine
FWHM	Full Width at Half Maximum
TEM	Transmission Electron Microscope
SEM	Scanning Electron Microscope

## Appendix A

In this appendix, we detail the dipolar approximation for the open cavity composed by aa random arrangement of AuNSs considered as dipoles. The polarizability amplitude ***p****_i_*(*ω)* of the *i*-th AuNS is

(A1) $$p_{i}=\alpha \left(\omega \right)E_{i}^{ex}\left(\omega \right)$$

where ${Ε}_{i}^{ex}\left(\omega \right)$ is the external electric field amplitude experienced by the *i*-th particle. It is composed by the incident field ***E***_0_(***r****_i_*, *ω*) and the field radiated by the *N*-1 surroundings AuNSs. In the dipolar approximation, the polarizability of an AuNS is [20]

(A2) $$\alpha =\frac{\alpha_{0}}{1-\left(2/3\right)ik^{3}\alpha_{0}}$$

where *α*_0_ is the polarizability of the AuNS that, according to Mie theory, reads

(A3) $$\alpha =\frac{6\pi i\varepsilon_{h}\varepsilon_{0}}{k^{3}}\frac{\psi_{1}\left(kr_{2}\right)A-m_{2}{\psi^{\prime }}_{1}\left(kr_{2}\right)B}{\varsigma_{1}\left(kr_{2}\right)A-m_{2}{\varsigma^{\prime }}_{1}\left(kr_{2}\right)B}$$

where $A={\psi^{\prime }}_{1}\left(m_{2}kr_{2}\right)-C{\chi^{\prime }}_{1}\left(m_{2}kr_{2}\right)$, $B=\psi_{1}\left(m_{2}kr_{2}\right)-C\chi_{1}\left(m_{2}kr_{2}\right)$ and $C=\frac{m_{2}\psi_{1}\left(m_{2}kr_{1}\right){\psi^{\prime }}_{1}\left(m_{1}kr_{1}\right)-m_{1}{\psi^{\prime }}_{1}\left(m_{2}kr_{1}\right)\psi_{1}\left(m_{1}kr_{1}\right)}{m_{2}\chi_{1}\left(m_{2}kr_{1}\right){\psi^{\prime }}_{1}\left(m_{1}kr_{1}\right)-m_{1}\chi_{1}\left(m_{2}kr_{1}\right)\psi_{1}\left(m_{1}kr_{1}\right)}$, where the prime denotes first derivative of the function with respect to its argument, and with *ψ*, *ς*, *χ* denoting the Riccati-Bessel functions [31], and *m*_1_ = (*ε*_1_/*ε*_h_)^1/2^ and *m*_2_ = (*ε*_2_/*ε*_h_)^1/2^ are the core and shell relative refractive indexes. The dielectric core and metallic shell particles are characterized by *ε*_1_ = *ε_r_* and *ε*_2_ = *ε_m_*, where *ε_r_* is the relative permittivity of the chosen dielectric material and the metal permittivity *ε_m_* is described through the Drude model as

(A4) $$\varepsilon_{m}\left(\omega \right)=\varepsilon_{\infty }-\frac{\omega_{p}^{2}}{\omega \left(\omega +i\gamma \right)}$$

where *ω_p_* = 2.183 × 10^15^ s^−1^ is the free-electron plasma frequency, *γ =* 1.41 × 10^14^ rad⋅s*^−1^* is the relaxation rate and *ε*_∞_ is the high frequency permittivity determined by matching the experimental data in the visible region.

## Appendix B

QNMs are solutions of Maxwell equations that are associated with a complex eigenfrequency ${\tilde{\omega }}_{n}=\omega_{n}-i\gamma_{n}$, where the imagery part is a measure of energy leakage in the system, quantified by the quality factor *Q_n_* = *ω_n_*/2*γ_n_*. Localized QNMs represent the surface occupied by the hot-spot modes. Their spatial extent and their localization transition can be quantified by the so-called inverse participation ratio, *ζ*, that was originally introduced in the theory of Anderson localization [32]. Localized QNMs have an important property, i.e., at a given point ***r*** and a given frequency *ω*, the electric field is dominated essentially by one mode*,* and the probability to find more than one mode giving a high electric field is very small, since high values of *ζ* parameter correspond to large fluctuations of local density of states (LDOSs) [33]. Another advantage is that QNMs are described by the Green’s function, Equation (3), that is closely related to the LDOSs.

Since the plasmon resonances correspond to poles of Green’s function [21], near to such poles Green’s functions and consequently local fields become large. A complex function of such resonances can be easily calculated by identifying the positions of the corresponding poles in the complex plane composed by the frequency *ω* and by the spectral width *γ* ≅ −*∂_t_* ln*ω*. Under the condition *γ* > 0, it can be immediately to recognized that the amplitude of field increases with time. In addition, another source of amplification of plasmon enhancements is given by the condition to be *in-phase* inside the nanogaps (condition that correspond to hot spots), while the subsequent nanogaps could be *out-phased* showing a sort of dark spots. In this scenario, the light polarization plays a not negligible role. This is because plasmon resonances depend on the restoring force resulting from noncompensated AuNSs surface charges, this restoring force depends ultimately on the combination of the shape of the AuNSs topographical combination with illuminating light polarization.

## Appendix C

Another way to describe the *super-selection rules* induced by the SERS effects due to open cavity can be described as follows. The Raman signal emitted light at the detection point ***r***_0_ detected in the far field, $I\left(r_{0},\omega \right)\propto \langle E^{*}\left(r_{0},\omega \right)E\left(r_{0},\omega \right)\rangle$, can be considered as a spectral mixture, we are interested to outline its spectral distribution. By using the Wiener-Khintchine theorem, we can write detected intensity as

(A5) I(ω)=1πRe∫0∞dτ2ε0c〈E(r0,0)E*(r0,τ)〉eiωτ

The electrical fields are caused by the electric dipole moment of the molecule involved, which the radiating electric field at a distance *r* is

(A6) $$E=pe^{i\left(kr-\omega t\right)}\frac{\omega^{2}\sin\theta }{4\pi \varepsilon_{0}c^{2}r}$$

where *c* is the light speed and the electric dipole ***p*** is the summation of all the molecules located inside the area subject to enhanced electric field. If we combine this last expression with Equation (9) we obtain [34]

(A7) d2σdΩd(ℏω)=ω4|Γ(ω,θ)|2Iin8π3c3ε0ℏRe∫0∞dτeiωτ〈p(0)p*(τ)〉

The spatial resolution of the Raman signal is defined by Γ(*ω*, *θ*), of which spatial dimensions are essentially given by the enhanced electric fields, essentially defined by the open cavity QNM states as described before, while the spectral mixture is defined by the electric dipoles. The enhancement maps, that here we name SERS maps, can be built from Equations (3) and (16) once the xyz configuration of the AuNPs is experimentally known.

Another relevant physical consequence is that the Raman cross section, Equation (16), scales with the fourth power of the enhancement. This is because Raman scattering involves two steps: in the first step a photon is temporarily absorbed and the molecule goes into virtual state, in the second step a photon is emitted while the molecule goes back to the ground state, although to a different vibrational state. Both such rates are enhanced by a factor |Γ|^2^. Since this factor is defined by the spatial arrangement of the AuNSs, whose relative nano-gaps define the plasmonic properties of the open cavity, and then the enhancement factor is strongly spatially localized inside the nano-gaps generated by the AuNSs arrangement.
